# Transmission of *Leishmania infantum* by *Rhipicephalus sanguineus* (Acari: Ixodidae) in Dogs

**Published:** 2017

**Authors:** Alaleh RAKHSHANPOUR, Abdolali MALMASI, Mehdi MOHEBALI, Sedigheh NABIAN, Hossein MIRHENDI, Zabihollah ZAREI, Abdolhossein DALIMI, Anita MOHAMMADIHA, Behnaz AKHOUNDI, Amrollah AZARM

**Affiliations:** 1. Dept. of Internal Diseases, Faculty of Veterinary Medicine, University of Tehran, Tehran, Iran; 2. Dept. of Medical Parasitology and Mycology, School of Public Health, Tehran University of Medical Sciences, Tehran, Iran; 3. Center for Research of Endemic Parasites of Iran (CREPI), Tehran University of Medical Sciences, Tehran, Iran; 4. Dept. of Parasitology, Faculty of Veterinary Medicine, University of Tehran, Tehran, Iran; 5. Dept. of Medical Parasitology and Mycology, School of Medicine, Isfahan University of Medical Sciences, Isfahan, Iran; 6. Meshkin-Shahr Research Station, National Institute of Health Research, Tehran University of Medical Sciences, Tehran, Iran; 7. Dept. of Parasitology, Faculty of Medical Sciences, Tarbiat Modares University, Tehran, Iran; 8. Dept. of Medical Entomology, Faculty of Medical Sciences, Tarbiat Modares University, Tehran, Iran

**Keywords:** *Rhipicephalus sanguineus*, *Leishmania infantum*, Canine visceral leishmaniosis

## Abstract

**Background:**

*Rhipicephalus sanguineus* is the most widely distributed tick in the world, which is partly due to its biological flexibility and the global distribution of its major host, the domestic dog. In Mediterranean region it could be principal reservoir host for *Leishmania infantum,* usually transmitted by the phlebotomine sand flies. In this study, we evaluated the vector potential of *R. sanguineus* in transmitting *L. infantum* to uninfected dogs.

**Methods:**

During 2014, five dogs with clinical manifestations of canine visceral leishmaniasis (CVL), high anti-*Leishmania* antibody titers and tick infestation, were selected from CVL endemic areas (Tehran and Alborz provinces). At least, twenty live ticks were removed from each infected dog. After morphological identification, the ticks were divided into two groups; ticks belonging to the first group were dissected for parasitological examinations and semi-nested PCR assay, and those of the second group were selected for the transmission of CVL caused by *L. infantum* to uninfected dogs. Following tick infestation, all uninfected dogs were kept for 9 months and examined monthly for clinical and serological tests.

**Results:**

Nearly, 67% of ticks were infected by *L. infantum* using the semi-nested PCR. All other parasitological tests of ticks were negative. Clinical examinations and serological tests of the investigated dogs revealed negative results. Nested-PCR test results performed on splenic biopsy samples of dogs were also negative.

**Conclusion:**

*L. infantum*-positive *R. sanguineus* ticks were unable to transfer *L. infantum* from infected dogs to healthy ones. The detection of *L. infantum* DNA in ticks collected from naturally infected dogs by semi-nested PCR does not prove their vectorial competence.

## Introduction

Ticks are the most important vectors of pathogens including bacteria, viruses and some protozoa (1, 2). *Rhipicephalus sanguineus* is one of the notable vectors of many pathogens of dogs in which a considerable number of them exhibit a zoonotic role (3, 4). *R. sanguineus* is the most widely distributed tick in the world due to its biological flexibility and the global distribution of its major host-domestic dog (5). Characterized as a blood-sucking vector, ticks are able to harbor various types of microorganisms during feeding. However, this characteristic does not guarantee their capacity to transmit all harboring microorganisms. For example, the protozoan parasite *L. infantum* (*synonym*: *L. chagasi*), is a common parasite transmitted through carriers worldwide (6) and is often ingested by *R. sanguineus*. However, the possible transmission of *L. infantum* to susceptible dogs by *R. sanguineus* is poorly understood (7, 8).

CVL is a life-threatening disease caused by *L. infantum* (4). CVL is endemic in the northwestern and southern parts of Iran and its prevalence ranges from 14.2% to 17.4% (9). The zoonotic form of VL (ZVL) caused by *L. infantum* occurs sporadically in all geographical zones of Iran but is endemic in some parts of North East, North West and Southern areas of the country (10–13). In Iran, the disease is naturally maintained in a complex epidemiological cycle that may include both domestic and wild life reservoirs, including domestic dogs, foxes, jackals and wolves (13, 14).

*Leishmania* parasites are transmitted by *Phlebotomus* sand flies (*Diptera*: *Psychodidae*). However, other forms of transmission have been discussed, especially in some areas where reports of CVL are released in the absence of known hosts (15). Secondary mode of transmission may take place through dog bites (15, 16), transplacentally (17, 18), through sexual contact (18) and blood transfusion (19). Besides *Phlebotomus* sand flies, other arthropods (such as fleas and ticks) may act as potential vectors of *L. infantum* (7). Discovering other ways for transmission of *L. infantum* to dogs and humans is undoubtedly a significant issue.

The hypothesis of transmission of *Leishmania* parasites by *R. sanguineus* tick (*Acari: Ixodidae*) was emphasized many years ago (20, 21) and has been discussed in recent years (22). With regard to biological habits, fleas and ticks which their role has not been demonstrated as vectors of pathogens, similar to many species of sand fly *Phlebotomus*, may swallow *Leishmania* parasites during feeding(22).The aim of this study was to investigate the infection of *R. sanguineus* ticks collected from confirmed *L. infantum-*infected dogs, as well as their ability to transmit *L. infantum* to healthy dogs by parasitologic, serologic and molecular methods. Among the specific serological tests, DAT was found to be more specific (72%–100%), sensitive (92%–100%), and practical particularly in endemic areas of the world (14). Therefore, DAT was exploited in our study for serodiagnosis of *Leishmania* infection in dogs. Semi-nested PCR provides a rapid, sensitive and specific alternative to traditional techniques (Direct examination and culture). Moreover, diagnosis of *Leishmania* infection and species identification is done simultaneously. Traditional techniques commonly used for diagnosing leishmaniasis do not differentiate *Leishmania* species, and their sensitivity is lower than molecular techniques (23).

Therefore, semi-nested PCR was employed in this study to determine *L. infantum* infection in ticks and nested PCR in dogs.

## Materials and Methods

### Ethical approval

The study was approved by the Ethical Review Board of the Faculty of Veterinary Medicine, University of Tehran, where this project was approved and run.

### Collection and identification of ticks

Selection of CVL-positive dogs, which were concurrently infested with *R. sanguineus* ticks, took place between the months of Jun to Sep 2014. At first, a number of dogs which were clinically suspected to CVL were identified in the suburbs of Tehran and Alborz Provinces, designated as endemic areas for CVL (24). Using rapid serological kits (Sens PERT, VETALL Laboratories, Korea) and Direct Agglutination Test (DAT)(14, 25), five dogs with high levels of antibody titers (above 1:1280) and concurrently infested by ticks, were selected. At least 20 ticks were collected from each dog (more than 100 ticks). The ticks were transferred into capped tubes, and wet cotton balls were placed beside them (in order to provide the required moisture) and transferred to the Parasitology laboratory of School of Public health, University of Tehran. All ticks isolated from infected dogs were identified based on the diagnostic keys (26, 27). The collected ticks included *R. sanguineus* and *R. bursa*, from which 100 *R. sanguine*us ticks of both sexes were selected, and the remaining *R. bursa* ticks were excluded.

*R. sanguineus* ticks are small and have elongated body shape. They are usually inornate and have short palps.Eyes and festoons are present. Coxa I is deeply cleft and spiracular plates are comma-shaped in males. An identifying character for the brown dog tick is the hexagonal basis capituli (26, 27). Finally, the identified *R. sanguineus* ticks were divided into two groups at the laboratory. Ticks of the first group including all stages were transferred to tubes containing ethanol (5%) at a temperature of −20 °C for subsequent dissection and execution of PCR technique; ticks of the second group were maintained alive under suitable conditions of temperature and humidity for further transmission to healthy dogs.

### Parasitological examination of ticks

To observe the promastigotes of *L. infantum* parasites in the digestive tract, salivary glands, and ovaries of ticks, the ticks were dissected. For this purpose, a drop of melted wax was poured on a slide and ticks were immediately placed on it from the ventral surface. Using special tools of dissection, incisions were made on the lateral sides of the body of ticks. Then the digestive tract, salivary glands, and ovaries were removed and placed on separate slides (28). After fixation in methanol and staining with Giemsa, they were examined under the oil immersion lens (×100) for detecting *L. infantum.*

### *L. infantum* DNA extraction from ticks

Twelve (6 pairs) of ticks were crushed and mixed for performing six separate semi-nested PCR assays. To extract *L. infantum* DNA from ticks, genomic DNA extraction kit of Bio Neer Company (iNtRON) was used. LINR4, LIN17, and LIN19 primers were used (29, 30).

### Detecting L. infantum in ticks by PCR

Polymerase Chain Reaction (PCR) using Thermal Cycler device during 33 cycles (94 °C for 30 sec, 58 °C for 30 sec, 72 °C for 1 min and 72 °C for10 min (final extension) was performed, and the genomic DNA was identified using agarose gel 0.6% with Safe stain solution (29).

### Exposure of healthy dogs to L. infantum infected ticks

Ten alive ticks of the second group were transferred to each of the five healthy dogs, which were negative for *L. infantum* infection, by rapid kits and DAT tests. Initially, the dogs were sedated by a mixture of ketamine 10% (5–7 mg/kg, Alfasan Co., Netherland) and acepromazine 1% (0.05mg/kg, Alfasan Co., Netherland). Then, the right or left flanks were shaved, and special small bags made of cotton tissue were placed and sutured to the skin. Consequently, the ticks were placed inside the bags, and using a thin strip the bags were tightened and fixed; a healthy dog was kept as a negative control. The ticks immediately attached to the skin of dogs and as we followed them in next days many of them were still attached for a few days.

### Evaluation of tick-infested dogs

Dogs were kept for 9 months in separate indoor places at the Faculty of Veterinary Medicine University of Tehran. All dogs were clinically examined and serologically tested monthly using DAT tests (25). At the end of the study, all dogs were again serologically evaluated, and at the same time to detect any *L. infantum* amastigotes, parasitological and molecular tests of their spleen biopsy specimens taken through ultrasound biopsy guided needles were done. Initially, splenic tissue samples were parasitologically assessed under Giemsa staining methods and cultured in RPMI 1640 medium. The specimens were also investigated by a nested PCR method. DNA extraction was performed by Gene All (106–101 | Exgene Cell SV mini, 100) according to the manufacturer’s recommendations (23, 31).

## Results

### Findings of ticks’ dissection

A number of 38 adult ticks were dissected and 35 slides of samples from salivary glands, intestines and ovaries were prepared. No evidence of *L. infantum* promastigotes was found in the provided slides.

### Identification of L. infantum in ticks by semi nested-PCR

**Fig. 1:**
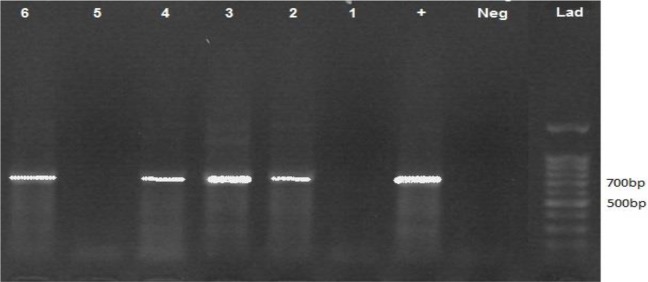
Positive results of ticks’ tissue samples in semi-nested PCR test

Gel analysis showed the infection in 4 of 6 pairs of crushed tick samples (66.6%) which undergone semi-nested PCR test (samples 2, 3, 4, 6) (Fig. 1). This finding confirmed the presence of *L. infantum* DNA in ticks collected from dogs with positive CVL infection.

### Post infestation serological tests of dogs

All those ticks attached to dogs were semi-engorged females. In dogs number 1–5, all ticks were detached after 5, 4, 7, 3 and 9 days, respectively. All clinical and serological examinations of dogs were negative during and after 9 months of trial.

### Parasitological and Molecular tests of dogs

**Fig. 2:**
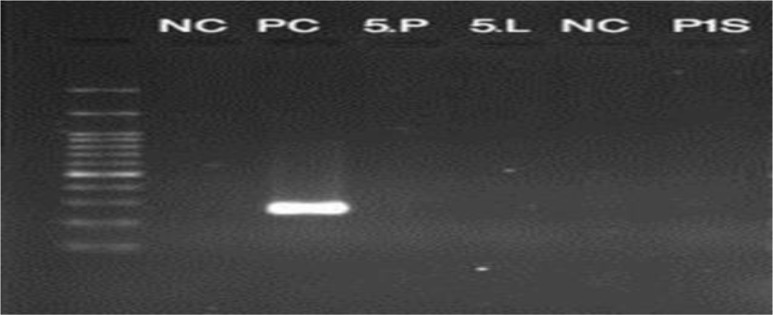
Splenic tissue samples showed negative results by nested PCR. Lane 1: 100bp marker; NC: Negative control; PC: positive control

None of the biopsy samples obtained from the spleen and stained with Giemsa, showed the presence of amastigotes. None of the five tissue biopsy specimens displayed any growth in RPMI 1640 culture medium after 7 days. Nested-PCR test results on biopsy samples were also negative (Fig. 2).

## Discussion

This study was performed for the first time to investigate the possible role of *R. sanguineus* in the transmission of *L. infantum* from CVL-confirmed dogs to healthy ones based on parasitological, serological and molecular methods. Considering the fact that ticks excrete a great amount of their imbibed water during blood feeding (22), the possibility that ticks could inject *Leishmania* parasites during blood feeding cannot be ruled out.

The natural infection of *R. sanguineus* is favored by many factors, including the high prevalence of both ectoparasite and protozoan in urban dogs within CVL endemic areas, the prolonged contact between ticks and dogs, the slow digestion of ticks and the substitution of hosts during their life cycle (32).

In the present study, no evidence of promastigotes or amastigotes of *L. infantum* was found in smears prepared from dissected ticks. The development of *Leishmania* in ticks could not be confirmed. Natural infection of *R. sanguineus* ticks by *Leishmania* like protozoa have been occasionally observed elsewhere (18). However, the presence of *Leishmania* – like protozoa in dissected ticks should be analyzed carefully. “Monogenetic trypanosomatids (e.g., *Leptomonas, Crithidia*, and *Blastocrithidia*) are known to infect ticks of several species including *R. sanguineus*“(8). These nonpathogenic trypanosomatids can be easily confounded with and misidentified as *Leishmania* parasites (8). Examination of slide smears is a less sensitive technique, and the relatively large volume of blood ingested by ticks makes detection of the parasites challenging.

PCR is a highly sensitive technique, allowing diagnosis of leishmaniasis by detection of parasite’s DNA. Small amounts of *Leishmania* DNA (≤ 1ng) can be detected in samples (32). In the study described here, tick colonies were incubated for 2 wk in order to assess the survival of *Leishmania* in the gut of *R. sanguineus*. When PCR was conducted to detect *L. infantum* in *R. sanguineus* ticks, an infection rate of 66.6% was obtained for the ticks examined. These results would indicate a higher infection rate by *L. infantum* in *R. sanguineus* ticks. As DNA of a few protozoa could show positive results, and as the procedure may detect fragments of the target DNA, results of PCR may be misleading. Hence, a positive PCR does not necessarily characterize a valid interactive infection of this protozoa with *R. sanguineus* (32). In agreement with the present study, *L. infantum* DNA was detected in *R. sanguineus* ticks in Brazil and Italy. In Brazil, the contamination percentage of ticks as 15.4 % was recognized (7). The contamination level of 27% in ticks was reported in Brazil (32). Contamination percentage of ticks had been reported as 47.16% in Meshkin Shahr region, Iran (33). In 2010, kinetoplast DNA (kDNA) of *L. infantum* was isolated from salivary glands of *R. sanguineus* ticks from infected dogs in southern Italy (8). A study was conducted on the possibility of transmission of leishmaniasis by infected ticks in the Golden Hamsters (*Mesocricetusauratus*), and a significant number of hamsters had positive results both in serology and molecular assays (7).

In the present study, *R. sanguineus* was susceptible to acquire *L. infantum* infection but not able to transmit it to the experimental canine host. Detection of *L. infantum* DNA in *R. sanguineus* ticks given their blood feeding habits, however does not confirm their vector potential. In a study by PCR technique, kinetoplast DNA (kDNA) of *L. infantum* in the larvae of *R. sanguineus* ticks were isolated after 4 months of experimental infection, which represents the possibility of trans-ovarial transmission of *Leishmania* (34). Earlier, Blanc et al., raised the possibility of transmission of *Leishmania* in trans-stadial mode in ticks (20).The results of Paz et al. investigations were consistent with the results of Blanc’s research (32).

Most existing theories about the transmission of *Leishmania* infection were reviewed and believed that ticks have no role in the transmission of VL in the Mediterranean region (35).

This study was carried out in accordance with the recent studies on the possible role of ticks in the transmission of CVL in Europe and South America (36).

In this study, dogs were infested by *L. infantum*-confirmed ticks, but no evidence of transmission ensued after 9 months of follow-up examinations. In 2010, in Brazil, some studies investigating the potential transmission of the agent of VL from ticks to hamsters showed positive findings. Coutinho, by releasing a documented evidence in this year, noted that he has managed to track *L. chagasi* infection in hamsters after 6 months (7). In Iran, no evidence of transmitting *L. infantum* to hamsters by infected ticks was reported (33).

## Conclusion

Data presented here provide further information on the role ticks might play in transmission of *L. infantum. L. infantum-*positive *R. sanguineus* ticks were unable to transfer the microorganism from infected to healthy dogs. The detection of *L. infantum* DNA in ticks collected from naturally infected dogs by semi-nested PCR did not prove their vectorial competence. However, PCR-based results showed a relatively high percentage of contamination with *L. infantum* DNA in dissected ticks. Further studies are needed to clarify the parasite behavior once inside the live tick tissues.
